# Oscillatory vapour shielding of liquid metal walls in nuclear fusion devices

**DOI:** 10.1038/s41467-017-00288-y

**Published:** 2017-08-04

**Authors:** G. G. van Eden, V. Kvon, M. C. M. van de Sanden, T. W. Morgan

**Affiliations:** 1 DIFFER—Dutch Institute for Fundamental Energy Research, De Zaale 20, 5612 AJ Eindhoven, The Netherlands; 2 Department of Applied Physics, Ghent University, St. Pietersnieuwstraat 41 B4, B-9000 Ghent, Belgium

## Abstract

Providing an efficacious plasma facing surface between the extreme plasma heat exhaust and the structural materials of nuclear fusion devices is a major challenge on the road to electricity production by fusion power plants. The performance of solid plasma facing surfaces may become critically reduced over time due to progressing damage accumulation. Liquid metals, however, are now gaining interest in solving the challenge of extreme heat flux hitting the reactor walls. A key advantage of liquid metals is the use of vapour shielding to reduce the plasma exhaust. Here we demonstrate that this phenomenon is oscillatory by nature. The dynamics of a Sn vapour cloud are investigated by exposing liquid Sn targets to H and He plasmas at heat fluxes greater than 5 MW m^−2^. The observations indicate the presence of a dynamic equilibrium between the plasma and liquid target ruled by recombinatory processes in the plasma, leading to an approximately stable surface temperature.

## Abstract (web summary)

Vapour shielding is one of the interesting mechanisms for reducing the heat load to plasma facing components in fusion reactors. Here the authors report on the observation of a dynamic equilibrium between the plasma and the divertor liquid Sn surface leading to an overall stable surface temperature.

## Introduction

Nuclear fusion power plants may turn out to be the sole candidate for centralised large-scale electricity production in a future carbon-free energy system. However, the largest obstacle on the development path of this technology is the tremendous power flux that hits the interior walls of such reactors. The largest fusion device to date, ITER (The Way in Latin), is currently being built and is expected to have a combined exhaust power from external heating and alpha particles of ≈150 MW^1^ while future electricity producing plants such as DEMO (DEMOnstration power plant) will have exhaust powers in the range ~580–~980 MW^2^. The latter device may possess an even narrower scrape-off layer width due to its larger size or increased magnetic fields^3^ hereby delivering a critical heat load to its exhaust area. The maximum heat load removal capability for conventionally designed divertors beyond ITER is not expected to increase much above the ITER limits of 5–10 MW m^−2^ ^4^ and the surface area receiving the power exhaust will remain similar to the case of ITER, which makes it essential to dissipate high-power fractions via radiation in the scrape-off layer and main chamber. As the tolerable heat load onto the divertor has a small error margin due to heat handling degradation for temperatures above recrystallisation such as observed for W^5, 6^, any accidental reduction of radiative cooling in DEMO and beyond causes increased divertor heat loads, which may be fatal to its armour integrity. At the same time, good divertor performance without regularly replacing its armour materials are essential for a fusion reactor to be commercially viable. Meeting such requirements using present day technologies are very challenging which makes investigating alternative divertor solutions a necessity.

The use of liquid plasma facing components (PFCs) can potentially alleviate many of the heat exhaust issues in the divertor. A liquid wall is self-healing as material displacement due to off-normal plasma impact is reversible. Lifetime issues related to erosion are less problematic because a liquid can replenish itself, which prevents damage accumulation, leading to a potentially longer lifetime. Additional heat transport by convective movement of the liquid, evaporative cooling^7^ and a reduction of neutron issues^8^ are other potentially beneficial properties of liquid PFCs. Finally and most importantly, when operating in the vapour shielding regime where a cloud of evaporated neutrals exists in front of the plasma-exposed surface^9, 10^, any accidental exhaust power excursion leads to increased evaporation, which may mitigate the impact on the divertor armour by self-protection. Despite these advantages, liquid metals are still at a low technology readiness level and require further development.

The effect of additional heat dissipation channels was recently demonstrated by the observation of a self-regulated heat flux mitigation phenomenon due to the presence of a Sn vapour cloud^11^. The upstream plasma heat flux was found to be almost completely decoupled from the average target surface temperature while the plasma temperature decreased to values close to 0.5 eV in front of the target surface. Although equilibrium timescale effects of the vapour presence were made clear in this study, the dynamical evolution and mechanism of the shielding phenomenon were not described.

Such questions have now been addressed and outcomes are reported here. The response of liquid Sn targets exposed to H or He plasmas in the power flux range of *q*_ref_ = 0.5–22 MW m^−2^ have been investigated. Conditions were chosen such that the Sn vapour pressure was of similar magnitude as the plasma pressure^11^. Solid Mo targets without vapour cloud formation were consequently exposed to equivalent plasma conditions, thus serving as a reference case.

The key result is that, during steady-state vapour shielding, the width and extent from the surface of the Sn vapour cloud oscillates in time in correlation with the target surface temperature and Sn emission intensity. A periodically varying shielding effectiveness resulting in a dynamic equilibrium between plasma and liquid surface is concluded. The obtained findings shed light on the dynamical aspects of steady-state vapour shielding by liquid metals at divertor-relevant plasma conditions. The outcomes of these investigations are pointing into a direction where the divertor-strike points are equipped with liquid metal technology (Sn, Li) while operated in a regime where the vapour pressure is of similar magnitude as the plasma pressure. If realized, steady-state operation of a liquid fusion reactor divertor is likely to be feasible.

## Results

### Surface temperature oscillations

**Fig. 1:**
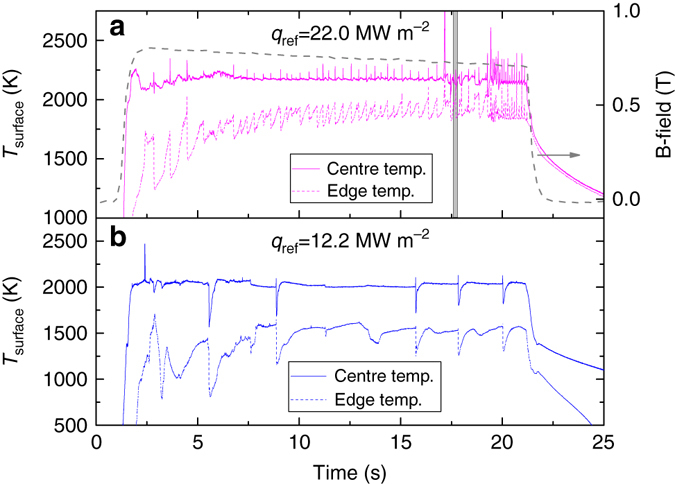
Evolution of the liquid Sn surface temperature. The temperature at the target centre and edge while exposed to a 22 (**a**) and 12.2 MW m^−2^ (**b**) He plasma as recorded by a fast IR camera. Fast temperature oscillations around a constant base value are regularly observed during discharges. The magnetic field strength over time is shown in a by the *right axis* and is identical for both *q*_ref_. The *grey box* indicates a temporal range which is analysed in detail in Figs. 2 and 3. The discharge at 12.2 MW m^−2^ shows slowly evolving temperature changes at larger time intervals

Our previous work^11^ reported on the striking difference between the thermal response of liquid Sn vs. solid Mo when exposed to equal plasma heat fluxes: the surface temperature at the end of the plasma discharge in the case of Sn was found to remain approximately constant while that of Mo continuously rose in accordance with increasing *q*_ref_. Another difference in the thermal response by these materials is evidenced by looking at the surface temperature over the course of the plasma discharge. Clear oscillations in surface temperature with an amplitude up to 200 K and period of roughly 100 ms are regularly observed at both the edge and centre of the Sn target, while the temperature response of the Mo target remains approximately constant once thermal equilibrium is reached. An example of this oscillatory behaviour is given in Fig. 1, which shows the temperature evolution in both the centre and at the edge of the target during He discharges at *q*_ref_ = 22.0 and 12.2 MW m^−2^.

**Fig. 2:**
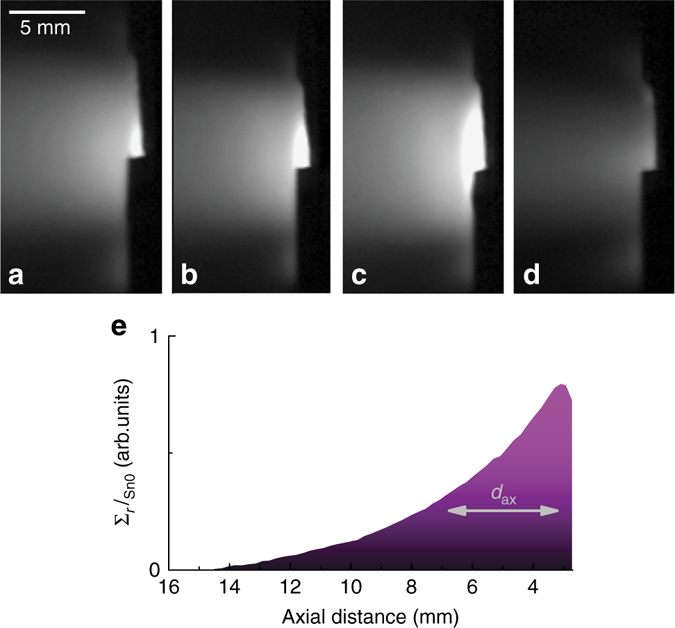
Oscillatory emission intensity from neutral Sn. A sequence of characteristic images during a single vapour shielding cycle as obtained from neutral Sn emission. The timestamps of the images **a**–**d** are 17.6, 17.64, 17.71 and 17.75 s after initiating a discharge at 22 MW m^−2^. The decay of Sn0 emission vs. distance from the target (*t* = 17.54 s) is shown in **e**

**Fig. 3:**
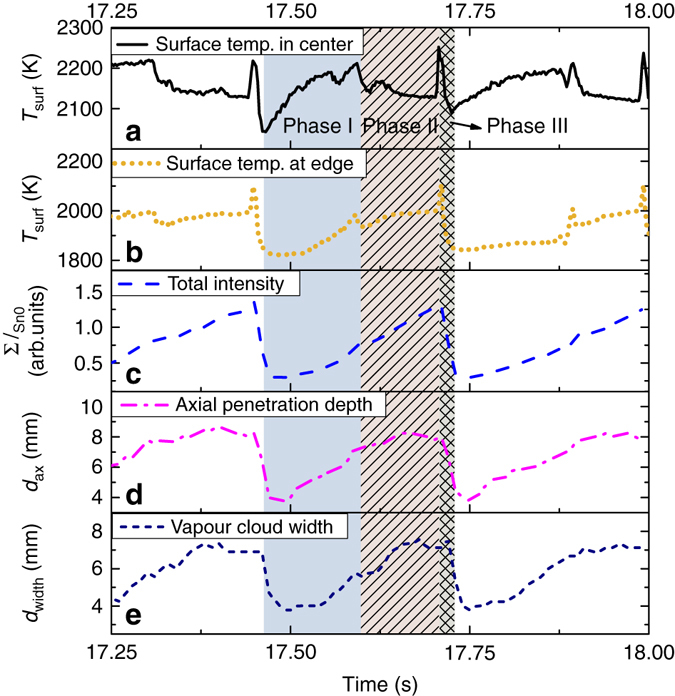
Vapour shielding dynamics. The surface temperature at the target centre (**a**) and edge (**b**) and Sn emission **c**–**e** during 17.2–18 s after initiating a 22.0 MW m^−2^ He discharge. Three characteristic phases within a typical oscillation period are indicated by the roman numerals I–III. **c** The total line-integrated intensity at 425.5 nm and **d**, **e** show, respectively, the penetration into the upstream plasma and the radial extent of the vapour cloud

The magnetic field strength is plotted vs. time in Fig. 1a. This dominantly sets *q*_ref_, which is thus seen to be approximately constant over the discharge duration. The edge temperature is seen to gradually increase followed by a rapid drop over the course of a single oscillation period while the temperature changes in the centre are much smaller during this period but are seen to rise rapidly at the end of each cycle. The *grey box* in Fig. 2a shows the period which is analysed in detail in Figs. 2 and 3. Figure 1b shows oscillations in the surface temperature, which are less regularly spaced but of much larger amplitude and temporal extent. This particular discharge therefore proved to be very suitable for analysis using diagnostics that have a time response which is usually too slow to observe fast fluctuating signals, such as target potential and spectroscopic measurements.

### Emission from neutral Sn

Recording the intensity of a distinct neutral Sn transition (*I*_Sn0_) at 452.5 nm (5*s* ^2^5*p*6*s*–5*s* ^2^5*p* ^2^) using the tangentially positioned fast camera allowed for investigating the vapour dynamics with high temporal resolution. A sequence of characteristic frames within a single oscillation cycle during a discharge at 22 MW m^−2^ is shown in Fig. 2. Frames in Fig. 2a–c qualitatively show the growth of the emissive region and magnitude of emission, which is the phase of continuous evaporation of Sn. Figure 2d shows the quenching of the plasma due to the high Sn impurity presence and is discussed later.

The neutral Sn emission is proportional to the product of the Sn atomic density *n*_Sn0_, the electron density *n*_e_ and the electron excitation rate coefficient *C*_exc_(*T*_e_):

1 $${I_{{{\rm Sn0}}}} \propto {n_{{{\rm Sn0}}}}{n_{{\rm e}}}{C_{{{\rm exc}}}}\left( {{T_{{\rm e}}}} \right).$$

Excitation from Sn0 to this particular excited state Sn0^*^ (5*s* ^2^5*p*6*s*) is much larger than the combined recombination rates from Sn^+1^ to Sn0^*^ at temperatures 0.5–0.8 eV^11^, even when taking into account that a large fraction of Sn is ionised^12^. This makes *I*_Sn0_ in Eq. (1) suitable for qualitative investigation of the Sn0 density.

Figure 2e shows $\mathop {\sum}\nolimits_r {{I_{{{\rm Sn0}}}}}$ (integrated over the beam radius) as a function of axial distance from the target. Given its exponential-like distribution, the ratio of the local intensity $\mathop {\sum}\nolimits_r {{I_{{{\rm Sn0}}}}(x)}$ and the maximum intensity $\mathop {\sum}\nolimits_r {{I_{{{\rm Sn0}}}}(0)}$ can be expressed by

2 $$\frac{{\mathop {\sum}\nolimits_r {{I_{{{\rm Sn0}}}}(x)} }}{{\mathop {\sum}\nolimits_r {{I_{{{\rm Sn0}}}}(0)} }} = {e^{ - x/{d_{{{\rm ax}}}}}},$$

with the typical axial penetration length of the evaporated Sn neutrals given by *x* = *d*_ax_. This treatment was repeated similarly for the intensity parallel to the target surface, giving the typical width of the Sn vapour cloud (assuming axial symmetry).

Figure 3a, b shows both the surface temperature at the edge and centre of the target, respectively, while the neutral Sn emission characteristics during this period are shown in Fig. 3c–e. The intensity at 452.5 nm was line-integrated and summed over all pixels in the non-saturated part of the image and is denoted as ∑*I*_Sn0_. A comparison of Fig. 3a–c shows that the surface temperature oscillates in correlation with ∑*I*_Sn0_. Results of time-resolved *d*_ax_ and the vapour cloud width (radial e-fold length) are shown in Fig. 3d, e, respectively.

Three phases during each oscillation period can be identified when examining the information in Fig. 3. Phase I is defined as the phase where the surface temperature increases in accordance with increasing ∑*I*_Sn0_ (roughly half the oscillation duration). Second, at half the cycle period, the phase where the central surface temperature starts to decline while *d*_ax_ still slowly increases is called phase II. Interestingly, ∑*I*_Sn0_ keeps progressively rising throughout this phase together with the edge surface temperature, which naturally results in a flattened radial surface temperature distribution at the end of phase II relative to phase I. Finally, phase III indicates a sharp increase in surface temperature followed by a sudden drop, which characterises the end of the cycle. This rapid temperature excursion does not show up in parallel in the emission profile and will be discussed later.

Both *d*_ax_ and *d*_width_ are seen to oscillate in time with the same periodicity as the surface temperature. Interestingly, when the cloud extends ~7 mm into the plasma (~100 ms into phase I) the surface temperature of the centre starts to decrease (entering phase II). The Sn vapour cloud extends further upstream in the remainder of the cycle and its width increases as indicated by the increasing e-folding lengths. The increase in *d*_width_ is in agreement with the continuously rising edge surface temperature. Since *d*_ax_ ∝ 1/*σn*_e_, where *σ* represents the collision cross section which is proportional to *T*_e_, a decrease of *T*_e_ and/or *n*_e_ is implied in this region. We thus reason that the static plasma pressure, *p* = 2*n*_e_ *kT*_e_ (assuming *T*_e_ = *T*_i_), periodically decreases as a result of interactions with the Sn cloud. This conclusion is in accordance with increased recombination as previously reported^11^. It is further concluded that the point at which the target temperature starts to decrease is set by the Sn vapour density depending on *q*_ref_, which is the period where effective shielding occurs (phase II).

### Plasma sheath potential

The floating potential *V*_fl_ of the Sn and Mo targets during the plasma discharges is measured at a time resolution of 250 ms. *V*_fl_ is the sum of the plasma potential *V*_p_, the sheath potential *V*_sh_ and pre-sheath *V*_ps_ potential relative to the ground: *V*_fl_ = *V*_p_ + *V*_sh_ + *V*_ps_. As the upstream plasma conditions and source behaviour are found not to change when switching between Sn and Mo targets, *V*_p_ is assumed to remain equal as well. Therefore, since *V*_sh_ ≈ 2.5*k*_B_ *T*_e_/*e* relative to *V*_p_^13^, measuring the floating potential provides an indirect method of investigating *T*_e_.

**Fig. 4:**
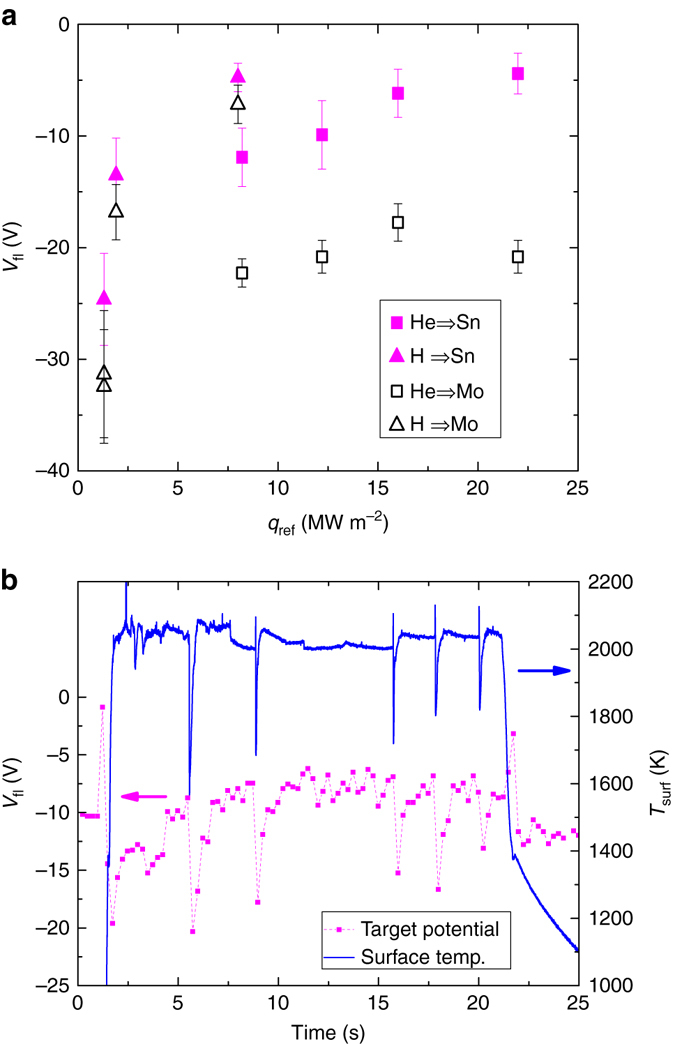
Changes in target potential during Sn vapour shielding. The average target floating potential in discharges on Mo and Sn (**a**) and temporal changes of the target floating potential measured during a 12.2 MW m^−2^ He discharge on liquid Sn in comparison to the Sn surface temperature evolution (**b**). *V*_fl_ in **a** is obtained from averaging the target floating potential over the range of constant magnetic field. The *error bars* represent the s.d. of these data sets

Figure 4a shows a comparison of the floating target potential during exposures of Mo and Sn targets averaged during the phase of constant magnetic field (3–21 s after initiating the discharge). A less negative target potential by a factor >2 is consequently observed for He exposures on liquid Sn and the effect seems to increase at larger *q*_ref_. The effect is small but also observable for the lower heat flux discharges using H. The relative increase in sheath potential when comparing the Sn and Mo exposures (*V*_p_ remains the same) at fixed *q*_ref_ directly correlates to reduced *T*_e_ in the case of Sn as obtained from Boltzmann plots made during the same discharges^11^.

Due to temporal constraints associated with these measurements, only the relatively slow oscillations that occurred during the 12.2 MW m^−2^ discharge could be well studied. Figure 4b shows both the surface temperature and time-resolved floating potential of this discharge for comparison. As can be seen, *V*_fl_ ≈ −10 V during the non-oscillatory phases 4–21 s after the start of the discharge. *V*_fl_ decreases however maximally to values ranging −20 to −15 V during the last phase of the vapour shielding cycle where the surface temperature strongly decreases. The latter values are close to the floating potentials as measured during Mo exposures presented in Fig. 4a.

Given that the target floating potential correlates with *T*_e_, it is concluded that *T*_e_ reduces during phase II while temporarily increasing during the period marked by the end of phase III and the start of phase I. Cooling of the plasma by neutral-ion elastic collisions and subsequent ion-electron elastic collisions^14^ is previously interpreted as the mechanism^11^. This statement is in agreement with the behaviour of the floating potential as reported above: the increase in vapour emission (Fig. 3c) means increased neutral Sn density causing increased neutral-ion friction via elastic collisions. The plasma cools by ion-electron cooling which is reflected in a less negative target potential. Once the vapour cloud is lost, *T*_e_ increases causing a more negative (Mo-like) target potential. Hence, also *T*_e_ is found to oscillate during the vapour shielding cycle.

### Continuum radiation

Emission spectra in the range of 360–580 nm in the near-surface region have been recorded. The continuum emission, clearly observable between the characteristic line emission features, emerges due to a combination of Bremsstrahlung and recombination radiation. It can be expressed as a simplified proportionality in the following way:

3 $${\epsilon _{{{\rm cont}}}} \propto \mathop {\sum}\limits_i {\frac{{{n_{{\rm i}}}{n_{{\rm e}}}}}{{\sqrt {{T_{{\rm e}}}} }} = \frac{{{n_{{\rm e}}}}}{{\sqrt {{T_{{\rm e}}}} }}\left( {{n_{{{\rm H}}{{{\rm e}}^{{\rm  + }}}}} + {n_{{{\rm S}}{{{\rm n}}^{{\rm  + }}}}}} \right) = \frac{{n_{{\rm e}}^2}}{{\sqrt {{T_{{\rm e}}}} }} \left[ {{{\rm W}}{{{\rm m}}^{ - 3}}{{\rm s}}{{{\rm r}}^{ - 1}}{{\rm n}}{{{\rm m}}^{ - 1}}} \right]} ,$$

where the sum indicates a summation over all ionic species present in the plasma. More complicated factors of *T*_e_ that predominantly affect the shape of the continuum emission distribution rather than its absolute levels are neglected in the proportionality expressed in Eq. (3). We used the assumption that both the He and Sn species are only maximally singly ionised, which cancels the dependency on the effective charge via ${Z_i}^2$ which appears in the full expression of ${\epsilon _{{{\rm cont}}}}$ ^15^.

**Fig. 5:**
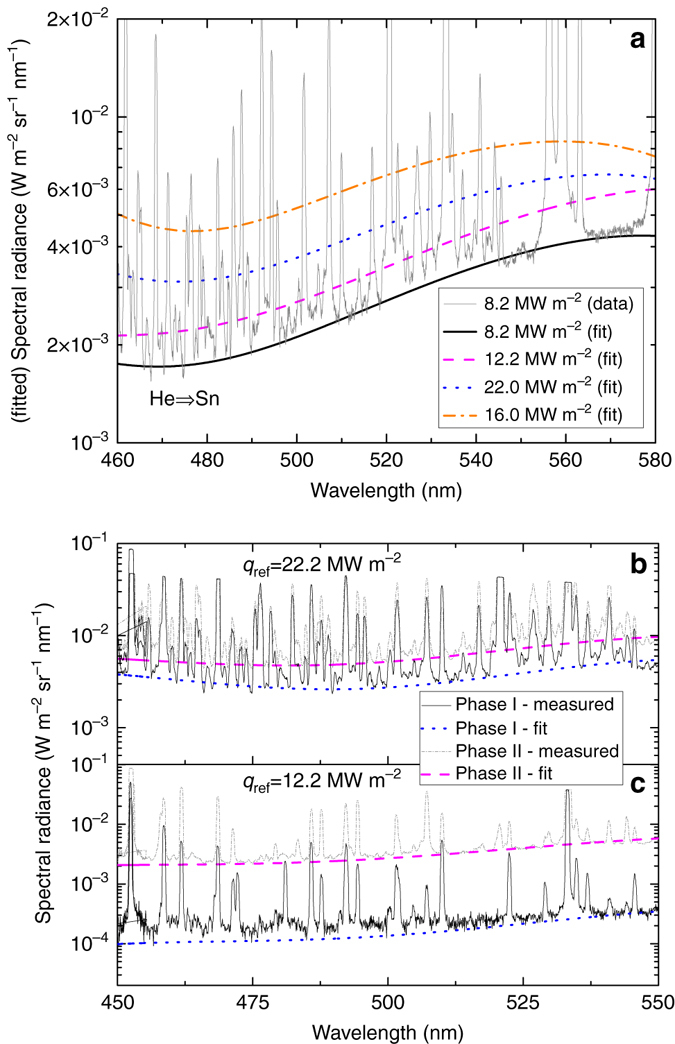
Oscillations in Sn-He plasma continuum emission. Polynomial fits representing the continuum emission acquired during measurements of the spectral radiance during He plasma exposures of Sn **a**. Experimental data of the discharge at 8.2 MW m^−2^ are shown for comparison. The spectral radiance of the near-surface plasma with a large fraction of Sn for discharges at *q*_ref_ = 22.2 MW m^−2^ (**b**) and *q*_ref_ = 12.2 MW m^−2^ **c**. The continuum emission (fits to the experimental data) of the near-surface plasma at the start of phase I and during phase II are shown by the *dotted blue lines* and *dashed red lines*, respectively

The spectral radiance during exposures of liquid Sn are measured and polynomial fits to the data are presented in Fig. 5a. When changing *q*_ref_ from 8.2 to 16.0 MW m^−2^, *n*_e_ changes from 4.1 × 10^20^ to 6.1 × 10^20^ m^−3^ (Table 1). Since *T*_e_ is found to be highly similar at ~0.6 eV for these discharges^11^, the increase in density should lead to an increase in the continuum emission by a factor (6.1/4.1)^2^ = 2.2 which is confirmed by the data shown in Fig. 5a.

**Table 1:** Experimental conditions during exposures of Mo and Sn targets

Gas	**B** (T)	*T*_e_ (eV)	*n*_e_ (×10^20^ m^−3^)	*q*_ref_ (MW m^−2^)
He	0.4	1.6	3.2	2.5
	0.8	2.4, 2.5, 2.7, 3.1	4.1, 5.5, 6.1, 7.0	8.2, 12.2, 16.0, 22.0
H	0.4	0.4	1.4	0.47
	0.8	1.2, 0.9, 0.9	0.6, 1.3, 1.5	1.3, 1.9, 8.0

The heat flux is expressed as the peak values from Gaussian fitted profiles of TS data. The gas flow was held constant at 2.5 slm while the plasma current was varied between 130 and 210 A

It follows from Eq. (3) that analysing the changes in continuum emission during a vapour shielding cycle provides insight in the evolution of the plasma parameters during the oscillation phase. Figure 5b, c shows the spectral radiance and polynomial fits to the data of He discharges at 22.2 and 12.2 MW m^−2^, respectively. Spectra at the start of phase I, where the surface temperature starts to increase but is still at a minimum, are now being compared to the spectra where vapour shielding most effectively occurs, namely halfway phase II where the central surface temperature is relatively constant or decreasing.

It is found that *n*_e_ changes by a factor $\sqrt {1.7}$ = 1.3 and $\sqrt {18.6}$ = 4.3 during the vapour shielding cycle for *q*_ref_ = 22.2 and 12.2 MW m^−2^, respectively. Recall that the emission from neutral Sn is found to steadily increase over the course of the vapour shielding cycle as shown in Fig. 3b. Also, despite the decrease of surface temperature in the centre during phase II, the edge temperature still rises (Fig. 3), implying a continuously increasing flux of Sn atoms released from the target. It is mentioned in ‘Emission from neutral Sn’ that the increase in mean free path of Sn atoms during the vapour shielding cycle as shown in Fig. 3c implied a reduction *n*_e_ and/or *T*_e_ in the centre of the plasma beam. However, from the increase of ${\epsilon _{{{\rm cont}}}}$ which is proportional to $n_{{\rm e}}^2$, an increase in electron density during the vapour shielding cycle is concluded. By realizing that the collision cross section of neutral Sn is highly sensitive on *T*_e_ while weakly dependent on *n*_e_, it is thus concluded that mean free path is increased due to reduction of *T*_e_ despite increasing *n*_e_. Hence, the observed increase in continuum radiation over the course of the vapour shielding cycle is explained by increasing *n*_e_ by a factor up to ~4 during this period. Since *T*_e_ is reduced by roughly the same factor when changing from a liquid to a solid target at equal upstream plasma conditions^11^, pressure (∝ *n*_e_ *T*_e_) along the plasma beam is conserved as expected.

### Liquid metal transport

**Fig. 6:**
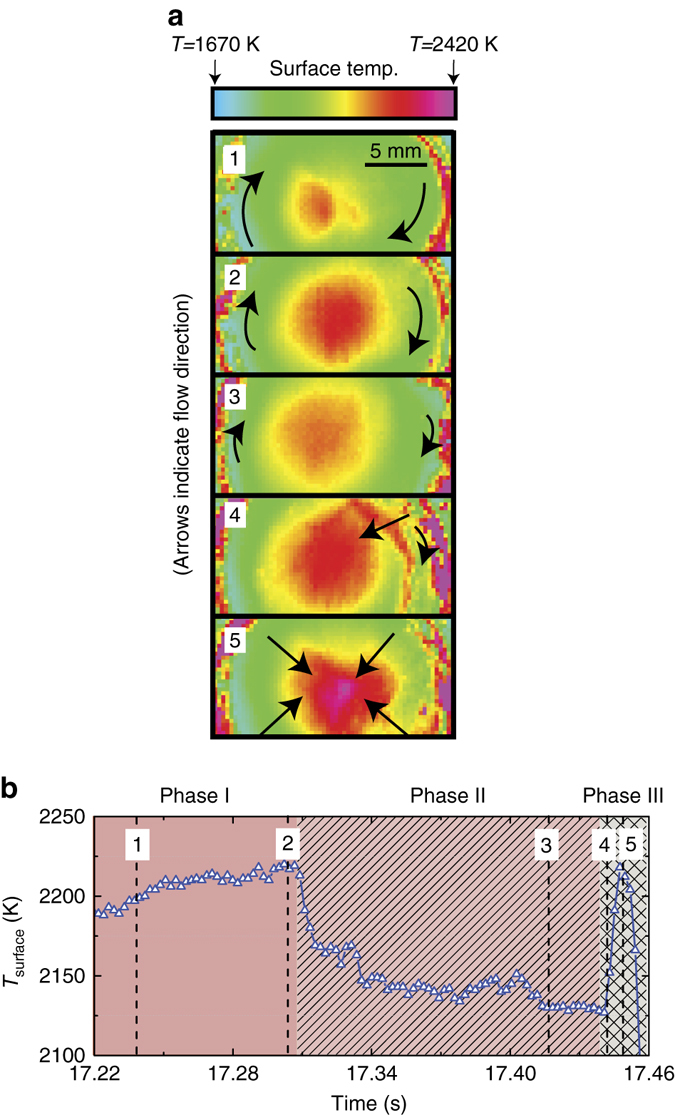
Flow dynamics of surface Sn during shielding cycle. A succession of IR images of the target surface during one vapour shielding cycle (**a**) and the temperature evolution during this cycle **b**. Time frames corresponding to the images are indicated by *vertical dashed lines* and labels. The *arrows* indicate the direction of the dominant surface flow while the sizes of the *arrows* qualitatively indicate the rotation speed

A thin layer of Sn is formed on the target surface during plasma exposure while the capillary porous system (CPS) secures the bulk liquid. This free liquid surface may give rise to convective flow, which influences the heat distribution along the target surface. Azimuthally directed liquid flow was observed during phases I and II of the vapour shielding cycle while phase III displayed a radial flow directed from edge to centre. A succession of IR images at five different times during one vapour shielding cycle, illustrating the different phases, are shown in Fig. 6. The IR images are linked to the temperature trace by the given numbers.

**Fig. 7:**
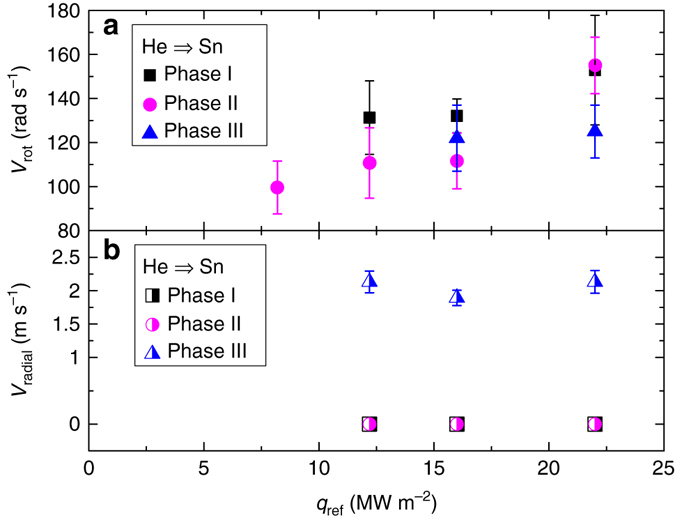
Liquid surface flow velocity. The velocity of rotating Sn at the edge of the target as function of *q*_ref_ for different stages during the oscillation cycle **a**. The rotational flow during phase II is seen to increase with *q*_ref_. A small decrease in rotation speed occurs during a single shielding cycle at heat fluxes of 12.2 and 16 MW m^−2^. A radial component to the flow velocity, only observed during phase III, is shown in **b**. The data points represent the average of ~10 independent measurements at equal phases within different vapour shielding cycles. The *error bars* represent the s.d. of these data sets

The speed of the liquid motion was quantified by monitoring the movement of emissive surface impurities in a succession of IR images similar to those shown in Fig. 6 and assuming that their speed equals that of pure Sn. Results of the average rotation speeds (*v*_rot_) and radial velocity (*v*_radial_) are shown in Fig. 7a, b, respectively. An increase in *v*_rot_ proportional to *q*_ref_ in case of rotation speed during phase II is observed. The rotational velocity as function of the phase within the oscillation cycle could only be analysed for discharges >10 MW m^−2^ since distinguishable impurities were found absent below this *q*_ref_. Although small, *v*_rot_ is seen to decrease in time when comparing phase I to phase II during discharges at 12.2 and 16.0 MW m^−2^. No changes in the liquid flow velocity were seen at highest *q*_ref_ when comparing between these phases. The rotation speed during phase III could only be measured for discharges at >16 MW m^−2^. When comparing phase III to the start of the cycle in the discharge at 22.2 MW m^−2^, a decrease in *v*_rot_ by 25% is observed. This effect is however not clear at 16.0 MW m^−2^. Furthermore, a radial component to the flow velocity, shown in Fig. 7b, is absent during phase I and phase II but rises however to ~2.1 m s^−1^ during phase III. The origins and magnitude of competing flow mechanisms as observed are discussed hereafter.

### Azimuthal flow

Liquid flow in a magnetic field (**B**) may arise as a result of Lorentz forces due to the presence of electric currents in the liquid. Since the flow was observed to rotate while **B** is directed into the plane of the target, a radial current must be present in the target. Both thermoelectric currents and externally injected current from the plasma column are potential candidates for this.

Radial currents are naturally occurring in the plasma column of Pilot-PSI as a result of its source potential configuration^16, 17^. It is clear from this work that there exists a net current carried by electrons in the centre of a floating target while the edge receives a net ion current. Such radial currents give rise to **J** × **B** driven rotation of the liquid^18^.

Since a radial temperature profile exists along the metallic interface comprised of the W mesh and liquid Sn, also a radial current (from centre to edge) in opposite direction of the thermal gradient arises as a result of so-called thermoelectric magneto-hydrodynamic (TEMHD) effect^19^. Following^20^, the thermoelectric current density *J*_TEMHD_ can be expressed as:

4 $${J_{{{\rm TEMHD}}}} = \frac{{\sigma P\nabla T}}{{C + 1}},$$

where *P* (=*P*_W_ − *P*_Sn_) represents the thermoelectric power of the solid–liquid pair and ∇*T* the thermal gradient along their interface. *C* denotes a non-dimensional thermal impedance ratio between liquid and solid and is calculated as *C* = *σ*_*S*n_ *h*/*σ*_W_ *t*_w_ where *σ*_Sn_ and *σ*_W_ represent the electric conductivity of Sn and W, respectively, *h* the liquid layer thickness and *t*_w_ the thickness of the W mesh. Given the directions of the aforementioned currents, the residual current density in the target *J* can be expressed as *J* = *J*_p_ − *J*_TEMHD_ with *J*_p_ the current density injected by the plasma. The rotation speed (*v*_rot_) as measured can now be used to calculate the incident current density from the plasma^20^:

5 $${J_{{\rm p}}} = {v_{{{\rm rot}}}}\sigma B{\left[ {1 - \frac{1}{{{\rm cosh}}({\rm Ha)}}} \right]^{ - 1}} + {J_{{{\rm TEMHD}}}}$$

with *σ* = *σS*_n_ and Ha the dimensionless Hartmann number describing the ratio of electromagnetic forces to viscous forces in a liquid. The Hartmann number is defined as ${{\rm Ha}} = Bh\sqrt {\sigma {{\rm /}}\mu }$ with *μ* the dynamic viscosity of the liquid.

Equations (4) and (5) are now evaluated for the case of the 22.0 MW m^−2^ discharge in He with *v*_rot_ = 140 ± 14 rad s^−1^. This yields a tangential velocity component of 1.4 ± 0.14 m s^−1^ at *r* = 10 mm, close to the target edge. A linearly decreasing surface temperature from edge to centre is assumed for simplicity. All parameters are evaluated at a temperature, which equals the average between the edge and centre as can be found in Fig. 3: *T*_av_ = (2050 + 1850)/2 = 1950 K and ∇*T* = (2150 − 1850)/0.01 = 2 × 10^4^ K m^−1^ (*r* = 10 mm) with a 10% error. Expressions for the temperature dependent thermoelectric powers of Sn and W up to 548 K are given in ref. ^21^. *P*_Sn_ above the Sn melting point is close to zero while *P*_W_ linearly rises at much higher values. We therefore estimate the relative thermoelectric power of Sn-W by assuming the value for W at 1950 K, which is 59.7 μV K^−1^. Given the large extrapolation, an error of 30% is assigned to the thermoelectric power. Values to the remaining variables in Eqs. (4) and (5) and the expression for *C* are assigned as follows: *B* = 0.8 ± 0.08 T, *σ*_Sn_ = 1.17 ± 0.12 × 10^6^ Ω^−1^ m^−1^, *σ*_W_ = 2.1 ± 0.21 × 10^6^ Ω^−1^ m^−1^, *h* = 5 ± 2.5 × 10^−4^ m, *t*_w_ = 5 ± 0.1 × 10^−5^ m, *μ* = 6.95 ± 0.7 × 10^−4^ Pa s, and, from this, Ha = 16.6 ± 5.2. The temperature-dependent electrical conductivity of W is calculated from the temperature-dependent thermal conductivity^22^ using the Wiedemann–Franz law. The error in liquid layer thickness is assumed to be 50%.

Substituting these numbers yield *J*_TEMHD_ = 20.7 ± 9.7 A cm^−2^ and *J*_p_ = 158 ± 105.9 A cm^−2^. We thus conclude that the dominant radial current is induced by the plasma rather then by thermoelectric effects. This is in agreement with the observed Azimuthal flow in clockwise direction. The weaker thermoelectric current flows in the opposite direction and would have caused counter-clockwise rotation if it were dominant.

### Radial flow

We secondly explore the radial surface transport. Since large temperature gradients exist across the liquid surface, surface tension-driven flows as described by the Marangoni effect may be present. The spatial temperature gradient is largest in the radial direction, which induces a radially outward surface tension driven flow. The treatment as provided in ref. ^23^ can be applied, giving the surface flow velocity as function of the tangential heat flux gradient with a normal incidence **B** as:

6 $${u_{{{\rm rad}}}} = \frac{{{{\rm d}}\gamma }}{{{{\rm d}}T}}\frac{{{h^2}}}{{\mu {k_{{{\rm Sn}}}}}}\frac{{\partial q}}{{\partial r}}\frac{{{{\rm sinh}}({{\rm Ha}})}}{{Ha \cdot {{\rm cosh}}({\rm Ha})}}$$

where d*γ*/d*T* is −0.14 m Nm^−1^ K^−1^, obtained from differentiating Eötvös law^24^. The liquid thermal conductivity is obtained by extrapolating a data set valid up to 1473 K^25^, providing *k*_Sn_ = 6.7 ± 0.7 Wm^−2^ K^−1^. The radial heat flux profile is measured by TS. A linearly decreasing heat flux over the target radius is now assumed for simplicity, estimated to be ∂*q*/∂*r* ≈ 5 ± 1/0.01 = 500 ± 100 MW m^−2^ m^−1^. All parameters are again evaluated at *T*_av_ = 1950 K resulting in *u*_rad_ = 0.23 ± 0.17 ms^−1^. Note that no radial flow in phases I and II could be observed (Fig. 7). It is however to be mentioned that the much faster Azimuthal flow may have impeded the observation of outward radial movement.

The ratio of convective to conductive heat transfer upon receiving a heat flux normal to the surface can be estimated as^23^:

7 $$\xi = {h^2}{u_{{{\rm rad}}}}\frac{{\rho {C_{{\rm s}}}}}{{{k_{{{\rm Sn}}}}}}$$

with *ρ* the Sn density and *C*_s_ the heat capacity equal to 6099 kg m^−3^ and 141.1 J kg^−1^ K^−1^, respectively^24^. Inserting furthermore the thickness of the liquid surface layer, its radial velocity (*u*_rad_) and length equal to the target radius one finds *ξ* = 0.74.

Since this ratio is close to 1, both convective and conductive heat transport are important. The fraction of heat transported by convection is thus not found to be dominant owing to its shallow depth and low flow velocity. It is furthermore hypothesised that the decrease of *v*_rot_ over the course of an oscillation period is caused by a reduction of plasma flux (mostly electrons) in the beam centre due to vapour shielding while the edge of the target continuous to receive heat flux (mostly ions). This is in agreement with the observation that the edge surface temperature rises during a cycle while the central temperature remains approximately constant. As a result of the effective shielding in the target centre, the sheath potential becomes less negative here (Fig. 4) while the edge sheath remains unaffected. The collapse of rotation and strong radial flow overall indicate that the plasma flux reaching the surface is strongly reduced during phase III, resulting in cool down of the target prior to a new cycle. Given that the plasma pressure is temporarily decreased as a result of detachment, we explain the inward movement in phase III by surface tension forces minimising the surface area after it is distorted by the pressure gradient imposed by the plasma. An inwardly spiralling flow in phase III results from this, despite the target centre being still hotter than the edge.

## Discussion

**Fig. 8:**
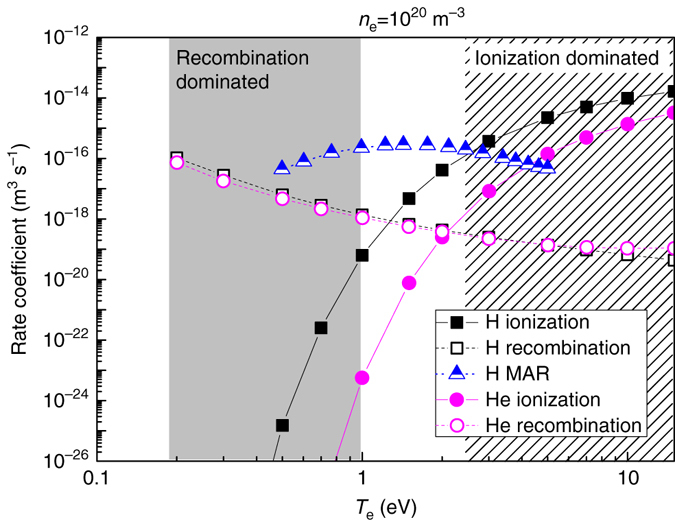
Ionisation and recombination rate coefficients of H and He. The coefficients, valid at *n*_e_ = 10^20^ m^−3^, are extracted from the ADAS database^26^. Molecular assisted rate (*MAR*) coefficients were obtained from ref. ^27^. The *shaded region* on the *right* highlights *T*_e_ values of a H and He plasma where ionisation is the dominant process. The *grey box* on the *left* indicates a recombination dominated temperature regime

The ionisation and recombination rate coefficients for H and He are shown as function of *T*_e_ for *n*_e_ = 10^20^ m^−3^ in Fig. 8. The data are extracted from ADAS^26^ while rates for molecular assisted recombination processes are obtained from ref. ^27^. Only a weak dependency on *n*_e_ is found for these numbers.

With help of these rate coefficients, we seek to explain the oscillatory vapour shielding by the following model. The evaporation of Sn during phases I and II of the shielding cycle progresses until a critical Sn density (depending on *q*_ref_) in the near-surface plasma is reached (Fig. 3). The plasma ions lose their energy by interaction with the neutral cloud followed by ion-electron cooling^14^. *T*_e_ reduces to values lower than 0.5–0.8 eV^11^, where strong recombination of the plasma occurs (Fig. 8), starting from the target centre where the neutral fraction is highest and stretching gradually to the edges during phase II. This recombination process affects the plasma in a positive feedback loop: recombination produces neutrals that further cool the plasma causing additional recombination. The result is a temporary detachment-like state of the plasma^28^ where *q*_ref_ is significantly reduced, combined with a small floating target potential relative to the plasma potential (low *T*_e_), and increased *n*_e_ as described in ‘Continuum radiation’. The rotation speed of the liquid film at the target surface is dominated by **J** × **B**-induced forces in phases I and II, which decreases over the course of the shielding cycle as the surface heat flux reduces. At the same time, the reduction of static plasma pressure (*P*_p_ = 2*n*_e_ *kT*_e_^13^) is highest in the target centre relative to the target edge. Hence, with reduced Azimuthal inertia of the liquid in phase III, the surface tension difference between centre and edge drives the liquid from the edge inwards (see ‘Radial flow’). When *q*_ref_ at the end of phase II is reduced due to plasma detachment, the surface starts to cool and the evaporation flux of Sn (an exponential function of temperature), quickly decreases. The vapour cloud almost completely disappears in phase III and the plasma thus reaches the target surface without strong interaction with the vapour (plasma is attached), hence the floating potential reaches values similar to that of a solid target. At this point, the plasma starts heating the surface again, which results in the common conduction-type heating curve as observed at the start of phase I. Consequently, a new vapour shielding cycle is initiated and the process repeats itself.

The oscillatory nature of the vapour shielding effect is thus understood to be a result of periodical plasma detachment induced by the high neutral Sn density in the near-surface plasma, constituting a mutually interacting system between *q*_ref_ and Sn evaporation. The oscillation frequency is observed to be roughly 10 Hz (Fig. 1) and likely to be driven by the characteristic thermal equilibration timescale, which is slow compared to the rapid detachment process. The timescale of the latter can be approximated by the ion-electron collision time: ${\tau _{{{\rm ie}}}} = {\tau _{{{\rm ei}}}} = {\tau _{{\rm e}}}{m_{{{\rm He}}}}{{\rm /}}2{m_{{\rm e}}} \simeq 0.2$μs at *T*_e_ = 0.8 eV and *n*_e_ = 10^20^ m^−3^ ^11^. A conservative estimate of the characteristic time for the vapour cloud to disappear in phase III can be estimated as ${\tau _{{\rm v}}} = {d_{{{\rm ax}}}}{{\rm /}}\sqrt {\left( {2{k_{{\rm B}}}{T_{{{\rm surf}}}}} \right){{\rm /}}m} \approx 16$μs when taking the thermal speed equal to the surface temperature at 1950 K. Therefore, on short timescales, while the plasma undergoes rapid cooling due to the runaway detachment process, the surface temperature rapidly decreases, as conduction to the coolant *q*_cond_ is approximately uniform and still high. This can be expressed by ${T_{{{\rm surf}}}}(t) = \left( {{T_0} - {T_{{{\rm cool}}}}} \right){e^{\left( { - t{{\rm /}}{\tau _{{\rm c}}}} \right)}}$, yielding ${\tau _{{\rm c}}} \simeq 250$μs (Fig. 3). The subsequent heating phase occurs however over a longer period due to the slower equilibration time for conduction, particularly at the plasma edge where the heating rate is relatively low so that *q*_cond_ ≈ *q*_ref_. The mismatch in the characteristic timescales (μs vs. ms) between thermal material processes and atomic physics taking place in the plasma is understood to be the ultimate cause of the oscillatory behaviour.

The rapid increase in surface temperature observed in phase III (spike) is discussed now. First, no transient increase in Sn0 emission is observed. Second, from inverting the 1D heat diffusion equation, an additional heat flux of 4 MW m^−2^ would be necessary on top of the existing *q*_ref_ to replicate the typical surface temperature increase as observed in Fig. 3. Given that such a transient additional heat flux is highly unlikely and such rapid heating/cooling unphysical, we regard the interpretation of a rapid temperature change erroneous. It is therefore likely to be a change in emissivity, which gives a false reading to the IR camera, possibly as a result of surface waves due to relaxation of the surface tension forces following the detachment phase (see ‘Radial flow’).

The scheme which we describe is applicable for a tokamak divertor region, where, if the neutral pressure (created by evaporation from a liquid metal and/or conventional detachment) is large enough, ion energies up to 10s of eV are likely to be reduced. Furthermore, **J** × **B**-driven flow would be directed in the radial direction of the machine. Since the magnitude and directionality of plasma-induced currents in a divertor could be highly different (and time-dependent) from the radial plasma-induced currents as observed in this study, the magnitude of TEMHD effects may become more important in such a geometry. The rotational flow as described in this paper is likely to be absent in a tokamak divertor due to the different orientation of **B**. Despite the differences in liquid metal flows between a tokamak and linear device, the oscillations are ultimately found to be induced by a detachment-like phenomenon of the plasma and differences in timescales between thermal equilibria of the liquid metal and the atomic physics taking place. The liquid flow and its time-varying nature affects the replenishment rate of the liquid surface and are therefore ultimately linked to liquid divertor design. A key parameter may be found in the effective heat conductivity between the liquid surface and cooled solid substrate, as this affects the cooling rate of the surface during the phase of efficient vapour shielding (phase II) and hereby the extent of the variation in surface temperature/evaporation during a cycle. Hence, it is implied from these arguments that the oscillatory vapour shielding phenomenon as described here is generic and not specific to the linear plasma geometry as used in the experiments.

Oscillations may be an indispensable mechanism for the heat flux dissipation by the liquid surface to be self-regulated. It is hypothesised that matching the vapour pressure to the plasma pressure^11^ is a key requirement to reach this regime. For the case of Sn, temperatures >1800 K are required, which can be reached during edge localised modes and disruptions in tokamaks. Steady-state operation might be possible using adequate substrate materials and strong baffling of the divertor to prevent excessive ingress of Sn to the main chamber, along the lines of a vapour box concept as developed for Li^29, 30^. However, the same regime could be reached for temperatures around 1000 K when using Li for which, by being low-Z, a much larger impurity fraction can be tolerated in the plasma, particularly under high-flux conditions where a high local redeposition is expected. The results as shown here using Sn may therefore be regarded as a proxy for Li experiments which are technologically more challenging due to the protected atmosphere required. Experimental investigations of steady-state Li vapour shielding are currently carried out and are expected to be reported soon.

Concluding, the Sn vapour/plasma system is found to oscillate around a stability point between plasma heat flux and surface temperature/evaporation, indicating the presence of a dynamical equilibrium set by the characteristic timescale of thermal processes in the liquid metal. The oscillations emerge from periodic changes between an attached plasma phase with strong evaporation of neutral Sn and a phase characterised by a detachment-like plasma culminating in a loss of the vapour cloud due to reduced evaporation. The oscillatory vapour shielding in response to a steady-state divertor plasma would also hold for a tokamak environment, given the nature of the processes and should therefore be considered in future liquid metal divertor designs. Regardless of the complicated oscillation mechanism, the results over the explored parameter range indicate oscillatory vapour shielding can have a significant reductive effect on the plasma power load received by the PFC^11^.

## Methods

### Linear plasma generator Pilot-PSI

**Fig. 9:**
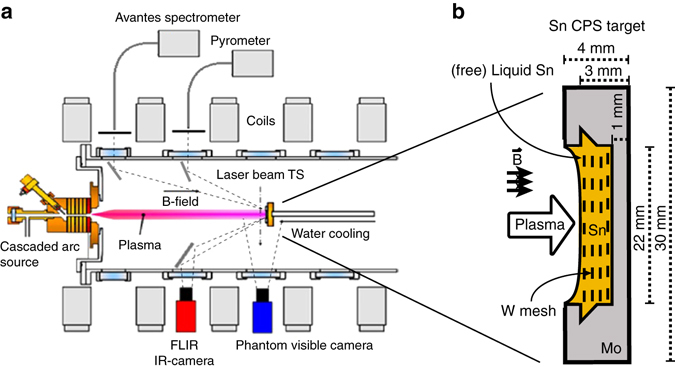
Schematic drawing of Pilot-PSI and liquid sample design. The linear plasma generator Pilot-PSI and the applied diagnostics are shown in **a**. Plasma is produced by a cascaded arc source. The expanding plasma is confined into a beam hitting the target by magnetic fields. A schematic drawing of the Sn CPS sample is shown in **b**

The experiments were performed in the linear plasma device Pilot-PSI, designed to study plasma wall interactions in ITER-like divertor regimes^31^ and is schematically shown in Fig. 9a. Although part of the results from this experimental campaign has been published before^11^, a description of the experimental setup is repeated here for completeness. Plasma in Pilot-PSI is produced by a cascaded arc source^32^ operating at a DC current ranging 130–210 A. Both H and He gas were flown into the source at 2.5 slm. By switching on an axial magnetic field up to 1.2 T, the plasma is confined into a beam hitting the target resulting in a typical particle flux of 5 × 10^24^ m^−2^ s^−1^. The electron temperature was 0.4–3.2 eV at densities 1–5 × 10^20^ m^−3^ in the centre of the plasma column. These values were obtained from Thomson scattering (TS)^33^, which measures the plasma parameters at a position of 11 mm in front of a solid Mo target.

### Diagnostics

The emission of Sn neutrals in the vapour cloud was recorded by a fast visible camera (Phantom V12) equipped with a 452.5 nm Sn0 filter positioned tangentially to the target surface. Recordings of the neutral cloud dynamics were made at 10 kHz temporal resolution. Spectroscopic information of the emitting cloud was obtained using an absolutely calibrated two-channel spectrometer (Avantes ULS2048), measuring photon intensities in the range of 360–580 nm. The system was aligned at a ~15° angle from the target normal and focused at the target centre with a spot size of ~1 mm. The resulting spectral intensities in Wm^−3^ sr^−1^ nm^−1^ are multiplied by the path length in the plasma of 0.02 m to yield the line-averaged spectral radiance in Wm^−2^ sr^−1^ nm^−1^. Time frames during a phase of (relatively) constant surface temperature were selected. H and He discharges below 8 MW m^−2^ are omitted as the continuum emission was found to be indistinguishable from the instrument noise. Finally, the surface temperature of the target was measured both using an infrared camera (FLIR SC7500MB) operated at 4.5 kHz and a multi-wavelength spectropyrometer (FAR associates FMPI). The latter provides an emissivity independent temperature measurement localised with a 1 mm diameter spot at the target centre, which is used to determine the emissivity of liquid Sn used by the IR camera.

### Liquid Sn sample technology

Splashing and ejection of liquid Sn into the plasma was limited by employing the so-called CPS in our sample manufacturing process^34^. This system for liquid metal containment has been tested on a number of tokamaks^35, 36^ and employs capillary action to resist gravity. The targets used in our experiments consist of a 3 mm deep Mo cup of 22 mm in diameter holding the liquid Sn, which is immersed in a stack of W meshes with a pore size of 0.2 mm. A schematic drawing of the sample, including the direction of the plasma and the magnetic field in our setup is shown in Fig. 9b.

### Exposure conditions

Strong evaporation was required to investigate the effect of the near-surface neutral Sn cloud on the power handling capabilities of the liquid target. A 4 mm thick Mo ring was placed behind the cup in order to reduce the heat conduction path between the target and the cooling structure due to the created interfaces. As such, a relative modest plasma power was found sufficient to yield a vapour cloud of similar vapour pressure as the plasma pressure^11^.

The particle- (Γ_part_) and heat flux (*q*) at the TS position were calculated from the radially resolved plasma density (*n*_e_) and electron temperature (*T*_e_) obtained from TS measurements during plasma exposure of the solid Mo target. The following equations have been applied^13^:

8 $${\Gamma _{{{\rm part}}}} = \frac{1}{2}{n_{{\rm e}}}\sqrt {k\left( {{T_{{\rm e}}} + \gamma {T_{{\rm i}}}} \right){{\rm /}}{m_{{\rm i}}}} $$

9 $$q = {\gamma _{{{\rm sh}}}}{k_{{\rm B}}}{T_{{\rm e}}}{\Gamma _{{{\rm part}}}}.$$

*T*_i_ ≈ *T*_e_ is assumed where *γ* = 5/3 (adiabatic flow with isotropic pressure). The ion mass (H or He) is represented by *m*_i_. The total sheath heat transmission coefficient (*γ*_sh_) in Eq. (9) is set equal to 7 again assuming *T*_e_ ≈ *T*_i_^13^. The particle- and heat flux profiles are typically Gaussian in Pilot-PSI. Consequently, a Gauss fit (FWHM 10.4 mm) is applied to the raw data and its peak value as given in Table 1 is used for analysis. The fractional errors of *n*_e_ and *T*_e_ are <6% and <7%, respectively for radial values ranging from −7 to 7 mm w.r.t. the centre of the plasma beam. Propagation of errors results in a fractional error of *q*_ref_ below 7%. Given that *q*_ref_ results from a fit, this error is an upper limit.

The upstream plasma heat flux (*q*) should be highly similar for both the solid and liquid target case as the penetration of Sn neutrals up to the TS position is negligible, (Fig. 1) and the current and voltage traces of the source are found to be independent of the target material. The TS measurements made on solid references therefore represent the upstream electron temperature and density in both the liquid and solid case, hence, *q* = *q*_ref._ Sn and Mo targets were also identically mounted ensuring equal conduction cooling properties.

### Data availability

All data that support the findings of this study are available from the corresponding author upon reasonable request.

## Electronic supplementary material

Peer Review file Reviewer reports and authors' response from the peer review of this Article at Nature Communications

## References

[1] Kallenbach A (2013). Impurity seeding for tokamak power exhaust: from present devices via ITER to DEMO. Plasma Phys. Control Fusion.

[2] Maisonnier D (2007). Power plant conceptual studies in Europe. Nucl. Fusion.

[3] Eich T (2013). Scaling of the tokamak near the scrape-off layer H-mode power width and implications for ITER. Nucl. Fusion.

[4] Rieth M (2013). Recent progress in research on tungsten materials for nuclear fusion applications in Europe. J. Nucl. Mater..

[5] Pitts RA (2013). A full tungsten divertor for ITER: physics issues and design status. J. Nucl. Mater..

[6] van Eden GG (2014). The effect of high-flux H plasma exposure with simultaneous transient heat loads on tungsten surface damage and power handling. Nucl. Fusion.

[7] Nagayama Y (2009). Liquid lithium divertor system for fusion reactor. Fusion Eng. Des..

[8] Mattas R (2000). ALPS-advanced limiter-divertor plasma-facing systems. Fusion Eng. Des..

[9] Gilligan J, Hahn D, Mohanti R (1989). Vapor shielding of surfaces subjected to high heat fluxes during a plasma disruption. J. Nucl. Mater..

[10] Sizyuk T, Hassanein A (2014). Scaling mechanisms of vapour/plasma shielding from laser-produced plasmas to magnetic fusion regimes. Nucl. Fusion.

[11] van Eden GG (2016). Self-regulated plasma heat flux mitigation due to liquid Sn vapor shielding. Phys. Rev. Lett..

[12] Hutchinson, I. H. *Principles of Plasma Diagnostics* 2nd edn (Cambridge University Press, 2001).

[13] Stangeby, P. *The Plasma Boundary of Magnetic Fusion Devices* 1st edn (IOP Publishing Ltd, 2000).

[14] Ohno N (2001). Static and dynamic behaviour of plasma detachment in the divertor simulator experiment NAGDIS-II. Nucl. Fusion.

[15] Muñoz-Burgos JM (2015). Applications of advanced kinetic collisional radiative modeling and Bremsstrahlung emission to quantitative impurity analysis on the National Spherical Torus Experiment. Phys. Plasmas.

[16] Shumack A, de Blank H, Westerhout J, van Rooij GJ (2012). Two-dimensional electric current effects on a magnetized plasma in contact with a surface. Plasma Phys. Control Fusion.

[17] Costin C, Anita V, Scholten J, De Temmerman G (2016). Tailoring the charged particle fluxes across the target surface of Magnum-PSI. Plasma Sources Sci. Technol..

[18] De Temmerman G, Daniels J, Bystrov K, van den Berg MA, Zielinski JJ (2013). Melt-layer motion and droplet ejection under divertor-relevant plasma conditions. Nucl. Fusion.

[19] Jaworski M (2010). Thermoelectric magnetohydrodynamic stirring of liquid metals. Phys. Rev. Lett..

[20] Jaworski M (2011). Macroscopic motion of liquid metal plasma facing components in a diverted plasma. J. Nucl. Mater..

[21] Fiflis P, Kirsch L, Andruczyk D, Curreli D, Ruzic DN (2013). Seebeck coefficient measurements on Li, Sn, Ta, Mo, and W. J. Nucl. Mater..

[22] Incropera, F. & Dewitt, D. *Fundamentals of Heat and Mass Transfer* 2nd edn (Wiley, 1985).

[23] Jaworski M, Morley NB, Ruzic DN (2009). Thermocapillary and thermoelectric effects in liquid lithium plasma facing components. J. Nucl. Mater..

[24] IIda, T. & Guthrie, R. I. *The Physical Properties of Liquid Metals* 1st edn (Clarendon Press Oxford, 1988).

[25] Yamasue E, Susa M, Fukuyama H, Nagata K (2003). Deviation from Wiedemann–Franz law for the thermal conductivity of liquid tin and lead at elevated temperature. Int. J. Thermophys..

[26] Summers, H. P. *The ADAS User Manual*. v2.6 http://www.adas.ac.uk (2004).

[27] Pigarov AY (2002). Collisional radiative kinetics of molecular assisted recombination in edge plasmas. Phys. Scr..

[28] Krasheninnikov SI, Kukushkin AS, Pshenov AA (2016). Divertor plasma detachment. Phys. Plasmas.

[29] Goldston RJ, Myers R, Schwartz J (2016). The lithium vapor box divertor. Phys. Scr..

[30] Goldston, R. J., Hakim, A., Hammett, G. W., Jaworski, M. A. & Schwartz, J. Recent advances towards a lithium vapor box divertor. *in press*. *Nucl. Mater. Energy* (2017).

[31] van Rooij G (2007). Extreme hydrogen plasma densities achieved in a linear plasma generator. Appl. Phys. Lett..

[32] Vijvers WAJ (2008). Optimization of the output and efficiency of a high power cascaded arc hydrogen plasma source. Phys. Plasmas.

[33] van der Meiden H (2008). High sensitivity imaging Thomson scattering for low temperature plasma. Rev. Sci. Instrum..

[34] Evtikhin V (1999). Calculation and experimental investigation of fusion reactor divertor plate and first wall protection by capillary-pore systems with lithium. J. Nucl. Mater..

[35] Mirnov S (2006). Experiments with lithium limiter on T-11M tokamak and applications of the lithium capillary-pore system in future fusion reactor devices. Plasma Phys. Control Fusion.

[36] Mirnov S (2009). Plasma-wall interactions and plasma behaviour in fusion devices with liquid lithium plasma facing components. J. Nucl. Mater..

