# Supplementary material for: Oscillatory vapour shielding of liquid metal walls in nuclear fusion devices
Source: Nat Commun. 2017 Aug 4;8:192. doi: 10.1038/s41467-017-00288-y (PMC5543134; doi:10.1038/s41467-017-00288-y)
Supplement: Supplementary file 1 — Reviewer reports and authors' response from the peer review of this Article at Nature Communications [file 41467_2017_288_MOESM1_ESM.pdf]

Reviewers' comments:

Reviewer #1 (Remarks to the Author):

This is an interesting and well written paper. The results are, as far as I can judge, based on reliable and accurate experiments. I support publication of the paper, but would suggest that the authors give more attention to discussing the physics (flows, heat transfer, and their effect on evaporation) of the liquid phase, which certainly plays a significant role. In particular, it would be interesting to see an estimate of the possible effect of the surface tension forces. At the size of the liquid target and the temperature gradients reported in the paper, the Marangoni effect must be quite strong.

There are a few typos in the text, and the reference to Fig. 6 on p. 10 needs to be fixed.

Reviewer #2 (Remarks to the Author):

Reviewer's report on

Vapour shielding of liquid metal walls in nuclear fusion devices: an oscillatory phenomenon

by

G.G. van Eden et al.

Extrapolation to DEMO of the strategies foreseen for power exhaust in ITER may turn out to be not obvious. Therefore, several advanced solutions are currently under study, among which the use of a liquid metal divertor.

This paper deals with the transient phase of a phenomenon which could be of interest for the understanding of the problem of power exhaust in future fusion reactors using liquid metal divertors. (The steady state was already discussed in Ref. 10.)

I think the paper could be considered for publication after a thorough revision, although I'm not sure if it is especially suitable for publication on a general audience journal like Nature Communications. Perhaps one of the other journals with multiple citations among the references of this paper, like Nuclear Fusion or Journal of Nuclear Materials, could be more suitable.

My general (major) comments are as follows:

1) I hardly see a reason why the present Section 3 should not change the title to Experimental setup, or similar, and become the first section after the (unnumbered and untitled?!) introduction, so that the Results become a bit more understandable to the reader.
2) The relevance of the paper for a tokamak configuration employing a liquid metal divertor, which is very different from the Pilot-PSI in many respects, is only briefly mentioned (lines 388-399), but deserves in my opinion a much more detailed discussion. For instance, as the authors themselves state that the Sn temperatures needed to match the plasma pressure may be too high for tokamak operation, I wonder why they did not use Li in the first place for their experiments. Other relevant differences are of course the B field direction with respect to the liquid metal surface, which will affect the sheath structure, and the extent of the heated zone (distance from center to edge), which will affect the temperature gradients.
3) In Section 1.5 the flow of the liquid metal on the target is discussed, but any discussion of convective heat transfer seems to be missing, as opposed to what implicitly promised at the beginning of the section (lines 234-235).

Other comments (in order of appearance in the paper):

a) In the introduction it should be important to separately discuss the effects of the scrape-off layer power e-folding length (λ_q) scaling from those of the actual size scaling, as only the latter is currently mentioned (line 25).
b) In figure 1b, please add edge data. In both 1a and 1b please add post-processed q(t).

c) In figure 2a-c the time corresponding to each frame should be specified. The print quality of this figure is rather poor.
d) In figure 3, please add post-processed q(t).
e) Please show in a figure the exponential decay of I_{Sn0} (line 115).
f) In figure 4, are the directions of the horizontal arrows correct?
g) In Section 3.3 it is mentioned that the liquid Sn is contained in a Mo cup. How is the cup replenished as the Sn progressively evaporates?
h) With reference to eq. (7), is any calibration available to assess the error bar in the estimated q?
i) In the references, I personally think it should be nice to always quote all of the authors, unless the Nature Comm. policy is against that, of course, which I don't know ...

Reviewer #3 (Remarks to the Author):

This is a well written paper based on substantial works. I recommend this paper for the publication. The main aim of this paper is about vapor shielding of liquid Sn under high plasma heat loads. Vapor shielding is a highly attracting subject within the fusion community and other field, for example, semiconductor processing. Plus, experiments with a liquid metal specimen are challenging and the authors successfully handle the experiment. Thus I believe that this work is very valuable and the publication of this article has a positive influence both for our community and the journal. However, some revisions should be taken before the publication in order to avoid misleading.

Section 1.2
How the surface temperature given in figure 3 is obtained? If this is from one-color IR images, how do the authors consider about effects of surface movement? A bumpy surface shows different optical emissions per apparent area compared with flat surface. Or some how CPS stabilize the surface movement??
In equation (1), effects of Sn ions are ignored. Sn neutral (Sn->Sn*->Sn) as well as recombining Sn ions (e-+Sn+ ->Sn* ->Sn) can emit the same radiation. The effect of Sn ions can be ignore for higher temperature where recombination processes have minor effects. However, in the lower temperature <1 eV as discussed in this paper, the recombination plays major roles.

Section 1.3
It is stated in the paper that the incoming plasma ion energy is cooled by neutral-ion friction and subsequent ion-electron cooling. It is not clear that this mechanism is limited for the low temperature (< 5eV) plasma flux or similar mechanism is possible for the reactor-relevant condition (~1 keV).
In addition, a sentence in page 7, "Cooling of the plasma by neutral-ion friction and subsequent ion-electron cooling [19] is previously concluded [10]" has several misleading points. First, the word "neutral-ion friction" should be replaced with "neutral-ion elastic collisions" or "neutral-ion friction via elastic collisions" as mentioned in ref [10]. "Friction" may mislead some of the readers. Second, it seems cooling by these reactions were not "concluded" but "interpreted" in ref. [10].
Legends of figure 4(b) does not agree with the arrows.

Section 1.5
Section 1.5 needs a major revision. It seems the main argument of this section is the MHD forced surface rotation is caused by plasma current and thermoelectric current is minor effect. I agree with this argument. However, it is better to state the flow of the logic apparently in the first part of this section.
In addition, it is better to include a discussion of the definition of the v_rot. I believe that it is natural to define the rotation speed in rad/s. If it is defined in m/s, the reason need to be provided.

Section 2
The detachment process of near wall plasma is discussed. The discussions in the first two paragraphs are about well-known detachment mechanisms and liquid metal motion as a consequence of heat flux and plasma current oscillation. There are still some questions remain. Why is the Sn layer disappeared so quickly after the detachment? (or is it reasonable speed?) and Where do these Sn particles disappear?

Discussion on the temperature spike in phase III need to be improved. Rapid cooling mechanism is a question as well as the heating mechanism. If the cooling timescale is unlikely short, the authors can conclude the spike is not actual surface temperature change.

Responses to the comments from the referees by van Eden et. al.

All responses from the authors can be found in Italic below.

Reviewer #1 (Remarks to the Author):

This is an interesting and well written paper. The results are, as far as I can judge, based on reliable and accurate experiments. I support publication of the paper, but would suggest that the authors give more attention to discussing the physics (flows, heat transfer, and their effect on evaporation) of the liquid phase, which certainly plays a significant role. In particular, it would be interesting to see an estimate of the possible effect of the surface tension forces. At the size of the liquid target and the temperature gradients reported in the paper, the Marangoni effect must be quite strong.

- *We thank the referee for his excellent remarks. Surface tension forces can indeed be large given the temperature gradients. We expanded section 1.5 dealing with liquid metal transport. We have included a new section which describes the magnitude of the radial flow caused by surface tension forces (the Marangoni effect). Although we do not clearly observe radial flow during phases I and II of the vapour shielding cycle, theoretically it should be present at around 0.2 m/s. Likely this is unobservable given the faster rotation speed and the lack of impurities in the centre of the target in these phases. Secondly, we have estimated the ratio of convective versus conductive heat transfer in the top liquid surface for our geometry which is close to 1. We have now included these calculations in the manuscript for a more complete description of liquid metal heat transport.*
- *Given the relatively slow velocity of liquid flow in the center of the target, we do not expect that the observed flows would change the evaporation rate. This is further reinforced by the observation that emission from neutral Sn, proportional to the evaporative flux, is found to be perfectly synchronized with the surface temperature oscillations.*

There are a few typos in the text, and the reference to Fig. 6 on p. 10 needs to be fixed.

- *Thank you for pointing out the error in the figure caption, this is fixed now. We have put our best efforts into correcting for typos.*

Reviewer #2 (Remarks to the Author)

Extrapolation to DEMO of the strategies foreseen for power exhaust in ITER may turn out to be not obvious. Therefore, several advanced solutions are currently under study, among which the use of a liquid metal divertor.

This paper deals with the transient phase of a phenomenon which could be of interest for the understanding of the problem of power exhaust in future fusion reactors using liquid metal divertors. (The steady state was already discussed in Ref. 10.)

I think the paper could be considered for publication after a thorough revision, although I'm not sure if it

is especially suitable for publication on a general audience journal like Nature Communications. Perhaps one of the other journals with multiple citations among the references of this paper, like Nuclear Fusion or Journal of Nuclear Materials, could be more suitable.

My general (major) comments are as follows:

1) I hardly see a reason why the present Section 3 should not change the title to Experimental setup, or similar, and become the first section after the (unnumbered and untitled?!) introduction, so that the Results become a bit more understandable to the reader.

- *We agree that the proposed order would make sense and is common in journals such as Nuclear Fusion. Positioning the methods section at the end of the paper is however common practice in the Nature Comms. style format. This is why we adhered to this policy. The same argument holds for the untitled introduction section.*

2) The relevance of the paper for a tokamak configuration employing a liquid metal divertor, which is very different from the Pilot-PSI in many respects, is only briefly mentioned (lines 388-399), but deserves in my opinion a much more detailed discussion. For instance, as the authors themselves state that the Sn temperatures needed to match the plasma pressure may be too high for tokamak operation, I wonder why they did not use Li in the first place for their experiments. Other relevant differences are of course the B field direction with respect to the liquid metal surface, which will affect the sheath structure, and the extent of the heated zone (distance from center to edge), which will affect the temperature gradients.

- *Using Li is an excellent suggestion. Studying Li vapour shielding has been a key focus in our research program. Since working with Li requires a protected atmosphere, manufacturing CPS samples and mounting them in Pilot-PSI required additional hardware which we did not have at the time. We decided to start experimenting with liquid Sn as a proxy for Li in vapour shielding studies. We have recently performed our first Li CPS experiments in Magnum-PSI, the successor of Pilot-PSI, with interesting results where vapour shielding oscillations at a divertor-relevant temperature of about 700 °C are clearly observed (data can be shown if requested). These promising results encourage us to continue our research and to publish this initial work using Sn.*
- *We agree with the referee that the section starting at line 388 is too succinct to deal with the differences in geometry. However, we would like to stress again that the direction of the magnetic field and electric currents in the target are likely to be of small or even non-existing influence on the oscillatory behaviour, as it is finally originating from a balance between liquid metal evaporation and resulting plasma recombination at widely different timescales. Bringing the vapour pressure equal to the plasma pressure is essential. The extend of the heated zone is also not largely influential. However, an important parameter is the heat transfer between the liquid surface and cooled solid substrate as this determines the cooling-rate of the surface temperature during efficient vapour shielding (phase II) and hereby the extend of the variation in surface temperature/evaporation.*
- *A few lines have been added to the manuscript in this section to clarify the previous points. Two sentences are added to the end of the section starting on line 400 related to Li experiments.*

3) In Section 1.5 the flow of the liquid metal on the target is discussed, but any discussion of convective heat transfer seems to be missing, as opposed to what implicitly promised at the

beginning of the section (lines 234-235).

- *We agree with the referee that the first sentence of section 1.5 was slightly misleading. We have removed this sentence. To expand the discussion of convective transport, we made an analysis of the surface tension driven flow (Marangoni) and the relative importance of conductive and convective heat transport in the liquid surface layer. This is now added to section 1.5.*

Other comments (in order of appearance in the paper):

a) In the introduction it should be important to separately discuss the effects of the scrape-off layer power e-folding length (λ_q) scaling from those of the actual size scaling, as only the latter is currently mentioned (line 25).

- *It is clear from works by Eich* (NF 53 (2013) 093031) *and Goldston* (NF 52 (2012) 013009) *that there is only a small dependency between λ_q and machine size. The scaling law is dominated by an inverse proportionality between λ_q and the poloidal magnetic field (B_p). However, for conventionally designed tokamaks such as ITER and DEMO, B_p is increasing with size which makes it an indirect size dependency (not to mention any turbulence effects that may be expected* (Halpern, NF 57 (2017))*. Since we are interested in divertor strike point heat loads for our studies, the power spreading factor is important. This factor is however difficult to assess for ITER and DEMO. The power entering the SOL - assuming a conventional aspect ratio tokamak - is simply strongly increasing as function of major radius. Hence, we chose to focus solely on this aspect for clarity.*

  We have added the following sentence to the first section in the introduction: "The latter device may possess an even narrower scrape-off layer width due to its larger size or increased magnetic fields (cite Eich) hereby delivering a critical heat load to its exhaust area."

b) In figure 1b, please add edge data. In both 1a and 1b please add post-processed q(t).

- *The edge temperature has been added. This is indeed a valuable addition.*
- *The plasma conditions are determined from averaging 30 Thomson Scattering recordings during 3 seconds halfway through the plasma discharge. Hence, q_{ref} is an average value which makes it impossible to plot this versus time as q(t). Using an average value of q is reasonable, as the upstream heat flux in Pilot-PSI is essentially constant over time (with a slight linear decay due to decreasing magnetic field strength over 10s of seconds). In order to track any changes in the upstream plasma power, we have checked the I-V characteristics of the source plates which were found unaffected by the vapour shielding oscillations (also stated in section 3.4). We have now added the magnetic field strength as function of time to figure 1, as this is proportional to the upstream heat flux.*

c) In figure 2a-c the time corresponding to each frame should be specified. The print quality of this figure is rather poor.

- *We thank the referee for this point. We have updated the images with timing marks and improved the print quality.*

d) In figure 3, please add post-processed q(t).

- *As stated in the response to comment (b), we have measured an average value of q_{ref}. Certainly over the short period shown in figure 3 this is constant and we therefore think that it is unnecessary to plot q(t).*

e) Please show in a figure the exponential decay of I_{Sn0} (line 115).

- *The exponential decay of I_{Sn0} is now included in figure 2 (panel e). We had to add this to the existing figure as the maximum number of 10 display items has been reached.*

f) In figure 4, are the directions of the horizontal arrows correct?

- *We thank the referee to point out this mistake. The errors were indeed mistakenly flipped and are corrected now.*

g) In Section 3.3 it is mentioned that the liquid Sn is contained in a Mo cup. How is the cup replenished as the Sn progressively evaporates?

- *The Mo cup is not actively refilled during consecutive exposures to plasma. Released Sn atoms are entrained in the strong plasma flux which is directed at the liquid surface. In addition, Sn atoms are easily ionized which makes these particles confined by the magnetic field in Pilot-PSI, hereby preventing radial outflow. These factors contribute to a very high redeposition fraction of evaporated Sn atoms. Previous studies, including but not only the study in ref. [11], pointed out redeposition fractions ≥87 % of evaporated Sn atoms. Additionally Sn from the edges/bottom of the cup is drawn to the surface by capillary action.*

h) With reference to eq. (7), is any calibration available to assess the error bar in the estimated q?

- *Mentioning the error in q is an excellent point. The Pilot-PSI Thomson Scattering system yields the plasma parameters (n_e and T_e) across the entire plasma column. The particle- and heat fluxes are calculated for each radial position using equations 6 and 7 in the manuscript resulting in a Gaussian-like shape. The values for q_{ref} as they appear in the manuscript are the maximum value of a Gauss fit. The fractional error for n_e and T_e are <6 % and <7 % for radial positions -7 < r < 7 mm respectively. These errors translate in a fractional error of maximally 7 % for the heat flux (q). Given that the data is subsequently fitted, this error should be an upper limit.*

  The following sentences have been added to section 3.4: "The fractional errors of n_e and T_e are <6% and <7% respectively for radial values ranging from -7 to 7 mm w.r.t. the centre of the plasma beam. Propagation of errors results in a fractional error of q_{ref} below 7%. Given that q_{ref} results from a fit, this error is an upper limit.

i) In the references, I personally think it should be nice to always quote all of the authors, unless the Nature Comm. policy is against that, of course, which I don't know ...

- *We have followed the advice as found in the Nature Comms. style guide: "All authors should be included in reference lists unless there are more than five, in which case only the first author should be given, followed by 'et al.'."*

Reviewer #3 (Remarks to the Author):

This is a well written paper based on substantial works. I recommend this paper for the publication. The main aim of this paper is about vapor shielding of liquid Sn under high plasma heat loads. Vapor shielding is a highly attracting subject within the fusion community and other field, for example, semiconductor processing. Plus, experiments with a liquid metal specimen are challenging and the authors successfully handle the experiment. Thus I believe that this work is very valuable and the publication of this article has a positive influence both for our community and the journal. However, some revisions should be taken before the publication in order to avoid misleading.

Section 1.2
How the surface temperature given in figure 3 is obtained? If this is from one-color IR images, how do the authors consider about effects of surface movement? A bumpy surface shows different optical emissions per apparent area compared with flat surface. Or somehow CPS stabilize the surface movement??
In equation (1), effects of Sn ions are ignored. Sn neutral (Sn->Sn*->Sn) as well as recombining Sn ions (e- +Sn+ ->Sn* ->Sn) can emit the same radiation. The effect of Sn ions can be ignore for higher temperature where recombination processes have minor effects. However, in the lower temperature <1 eV as discussed in this paper, the recombination plays major roles.

- *The surface temperature is obtained from IR images recorded in the range 3.97-4.01. A (emissivity independent) temperature measurement is obtained from a multi-wavelength pyrometer localized with a 1 mm diameter spot at the target centre, which is used to determine the emissivity used by the IR camera. Movement of the liquid is only seen at the edge of the target, where the liquid is pushed towards the edge of the cup (on top of the mesh) and can rotate freely. We believe that the liquid is barely moving in the center of the target during phases I and II because of the following reasons. Firstly, no moving impurities are observed. Secondly, emission from neutral Sn which is proportional to evaporation is found to be perfectly synchronized with the surface temperature oscillations. As changes in evaporation flux are an indirect indication of surface temperature variations, the role of emissivity changes can be regarded small or negligible.*

  However, we believe that the anomalous temperature variation during phase III may be a result of an emissivity change due to a surface wave (relaxation of surface tension forces). This is concluded as the temperature change is faster than based on conduction or convection: conduction based would require a sudden increase in heat flux of 4 MW/m^2 whereas convective transport would be too slow at this timescale due to viscous drag. It is possible that a disturbance of the surface (surface wave) causes the emissivity to change for a short time interval, hereby erroneously indicative of a high surface temperature. Finally, no corresponding transient increase in the evaporation is observed which is in line with the conclusion that the effect is not an actual temperature excursion. We mention this explicitly now in the discussion section.

- *We think that equation 1 gives a good approximation for the ground state density of Sn for the following reason. It is known from ref. [11] that T_e in the Sn vapour cloud is typically ~0.8 eV. Although the ionization rate from the neutral ground state is 10 times higher than the recombination rate into the neutral ground state at this temperature, the excitation rate associated with Sn0→Sn0* ($5s^25p6s$ → $5s^25p^2$, λ=452.5 nm) is ~600 times larger than the combined recombination rates from Sn^{+1} state to Sn0* ($5s^25p6s$). The ratio of excitation from the neutral ground state versus recombination from the ion level into the mentioned Sn0* level becomes smaller with decreasing T_e but the amount of ionized Sn rapidly falls close to zero (Kvon, submitted to NF, 2016). We can thus assume than the recorded emission at 452.5 nm represents the density of the neutral Sn ground state well. Previous calculations are obtained from Hutchinson, Principles of Plasma Diagnostics 2nd edition, formulae 6.3.3 and 6.3.27. Graphs can be send if wished.*

Section 1.3
It is stated in the paper that the incoming plasma ion energy is cooled by neutral-ion friction and subsequent ion-electron cooling. It is not clear that this mechanism is limited for the low temperature (< 5eV) plasma flux or similar mechanism is possible for the reactor-relevant condition (~1 keV).
In addition, a sentence in page 7, "Cooling of the plasma by neutral-ion friction and subsequent ion-electron cooling [19] is previously concluded [11]" has several misleading points. First, the word "neutral-ion friction" should be replaced with "neutral-ion elastic collisions" or "neutral-ion friction via elastic collisions" as mentioned in ref. [11]. "Friction" may mislead some of the readers. Second, it seems cooling by these reactions were not "concluded" but "interpreted" in ref. [11].
Legends of figure 4(b) does not agree with the arrows.

- *We thank the referee for these excellent remarks. The scheme which we describe is applicable for the divertor region, where, if the neutral pressure in the region (created by evaporation from a liquid metal and/or conventional detachment) is large enough, ion energies up to tens of eV are likely to be reduced. An alternative solution must be provided for fast ions.*
- *We will avoid using the word friction as this can indeed be a source of confusion. The sentence mentioned is changed into: "Cooling of the plasma by neutral-ion elastic collisions and subsequent ion-electron elastic collisions [19] is previously interpreted as the mechanism [11]."*
- *We have changed the arrows in 4(b) which were wrong indeed.*

Section 1.5
Section 1.5 needs a major revision. It seems the main argument of this section is the MHD forced surface rotation is caused by plasma current and thermoelectric current is minor effect. I agree with this argument. However, it is better to state the flow of the logic apparently in the first part of this section. In addition, it is better to include a discussion of the definition of the v_{rot}. I believe that it is natural to define the rotation speed in rad/s. If it is defined in m/s, the reason need to be provided.

- *We have expanded section 1.5 by including a theoretical description of surface tension driven flow (Marangoni) and the ratio of conductive versus convective flows. The structure of the first section of 1.5 was indeed unclear. We have tried to improve on this and added subsections to 1.5 in order to make the structure more clear.*
- *We have changed the figure to show the rotation velocity in rad/s. However, we calculate the radial plasma current from the rotational velocity using the Navier-Stokes equation for a conducting liquid, following the equation in ref [25], which takes the lateral flow maximum as an*

input expressed in m/s. Since we perform the calculation at 1.4 m/s in the following section, we added a line (near line #290) to convert rad/s to m/s.

Section 2

The detachment process of near wall plasma is discussed. The discussions in the first two paragraphs are about well-known detachment mechanisms and liquid metal motion as a consequence of heat flux and plasma current oscillation. There are still some questions remain. Why is the Sn layer disappeared so quickly after the detachment? (or is it reasonable speed?) and Where do these Sn particles disappear?

- *A large fraction of evaporated particles (>87 % [ref 11]) are redeposited onto the target surface. The remaining fraction leaves the plasma/target and condenses at the machine walls. The Sn cloud is basically in a steady-state balance between influx from the target via evaporation (source) and redeposition back on the target (sink). During detachment, the source term decreases exponentially due to the exponential relation between evaporation and surface temperature. Redeposition is a quick process: its characteristic timescale can be estimated as the average upstream penetration depth of 6 mm (fig. 3) divided by the thermal speed (taking the surface temperature at 1950 K): $t_c = d_{ax}/\sqrt{2\ k_B T/m}$ = 16 µs. Recombination followed by loss to the machine walls would yield similar numbers given that the plasma radius is of similar size as d_{ax}. Also, this estimation is conservative as the energy of the neutrals gained by the plasma is neglected. Despite the quick redeposition, the vapour cloud is seen to disappear (or rather decrease in size) after the detachment phase in a few milliseconds. This is thus likely to be driven by the cooling-down time of the surface, which temporarily decreases the source term. We have added this quick calculation to the manuscript in the discussion section.*

Discussion on the temperature spike in phase III need to be improved. Rapid cooling mechanism is a question as well as the heating mechanism. If the cooling timescale is unlikely short, the authors can conclude the spike is not actual surface temperature change.

- *We thank the referee for this good suggestion. We have now calculated the incident heat flux that would be necessary in addition to cause such a rapid temperature spike. The following numbers are obtained from figure 3: dT=124 C, dt=4 ms. By inverting the 1D heat diffusion equation, one obtains q_{spike} = 4 MW/m^2. We conclude that it is highly unlikely to transiently produce an additional heat flux (on top of the existing steady-state q_{ref}) of this magnitude. Further, no increase of Sn emission is seen as would be expected for such a large temperature increase. We therefore conclude that the 'thermal spike' is an emissivity effect. In phase III, after detachment, a relaxation process of the surface tensions forces causes the surface to reflow. It is highly possible that a surface wave causes a change in emissivity which is interpreted by the IR camera as a temperature change. We have significantly changed the section in the discussion describing phase III.*

REVIEWERS' COMMENTS:

Reviewer #1 (Remarks to the Author):

The authors have fully addressed all my comments. The manuscript can be published in its revised form.

Reviewer #2 (Remarks to the Author):

I believe the authors have properly addressed my previous comments, thank-you. I have no further comments.

Reviewer #3 (Remarks to the Author):

The authors considered my questions very seriously and gave reasonable answers. Now, I can recommend the abstract for publication. But before publication, I want to ask one last revision. The section title of 1.3 "Plasma sheath effects" is not appropriate. The section discusses about investigations of Te through the measurements of the plasma floating potential. Thus, it is better to be "Plasma sheath potential", "Plasma sheath oscillation", "Floating potential oscillation" or something else. After the revision, I believe this is an excellent paper which attracts interests from many researchers.

Responses to the comments from the referees by van Eden et. al.

All responses from the authors on the **resubmitted manuscript** can be found in Italic below.

Reviewer #1 (Remarks to the Author):

The authors have fully addressed all my comments. The manuscript can be published in its revised form.

Reviewer #2 (Remarks to the Author):

I believe the authors have properly addressed my previous comments, thank-you. I have no further comments.

Reviewer #3 (Remarks to the Author):

The authors considered my questions very seriously and gave reasonable answers. Now, I can recommend the abstract for publication. But before publication, I want to ask one last revision. The section title of 1.3 “Plasma sheath effects” is not appropriate. The section discusses about investigations of Te through the measurements of the plasma floating potential. Thus, it is better to be “Plasma sheath potential”, “Plasma sheath oscillation”, “Floating potential oscillation” or something else. After the revision, I believe this is an excellent paper which attracts interests from many researchers.

- *We appreciate the final suggestion from the referee. It is indeed true that we do not discuss changes in the plasma sheath in detail, but rather focus on measurable changes of the target floating potential. We have therefore followed the advice from the referee and changed the section title into ‘plasma sheath potential’.*
